# Diversity Takes Shape: Understanding the Mechanistic and Adaptive Basis of Bacterial Morphology

**DOI:** 10.1371/journal.pbio.1002565

**Published:** 2016-10-03

**Authors:** David T. Kysela, Amelia M. Randich, Paul D. Caccamo, Yves V. Brun

**Affiliations:** Department of Biology, Indiana University, Bloomington, Indiana, United States of America

## Abstract

The modern age of metagenomics has delivered unprecedented volumes of data describing the genetic and metabolic diversity of bacterial communities, but it has failed to provide information about coincident cellular morphologies. Much like metabolic and biosynthetic capabilities, morphology comprises a critical component of bacterial fitness, molded by natural selection into the many elaborate shapes observed across the bacterial domain. In this essay, we discuss the diversity of bacterial morphology and its implications for understanding both the mechanistic and the adaptive basis of morphogenesis. We consider how best to leverage genomic data and recent experimental developments in order to advance our understanding of bacterial shape and its functional importance.

## Abstract 2

The bacterial domain displays a spectacular diversity of cellular shape. This Essay explores our surprisingly limited understanding as to how and why these shapes have evolved and discusses ways in which this dearth of knowledge can be addressed.

## Introduction

Imagine a bacterium. Did you imagine asymmetric, multicellular filaments of curved bacteria, such as those belonging to the genus *Simonsiella* (Fig 1.21)? These bacteria glide slowly on the surface of your palate using the concave side of their curved cells and divide parallel to their long axis [1]. Or perhaps instead you imagined a photosynthetic, ovoid bacterium like *Rhodomicrobium vannielii*, which grows extensions of the inner membrane, cell wall, and outer membrane (Fig 1.19)? From its elongating (and sometimes branching) extensions, this bacterium can bud one of three types of cells: appendaged cells like itself; swimming cells; or angular, heat-resistant exospores [2]. The appendaged daughter cells, remaining attached to the mother cell, grow their own extensions and progeny to create giant networks of connected cells in the mud.

**Fig 1:**
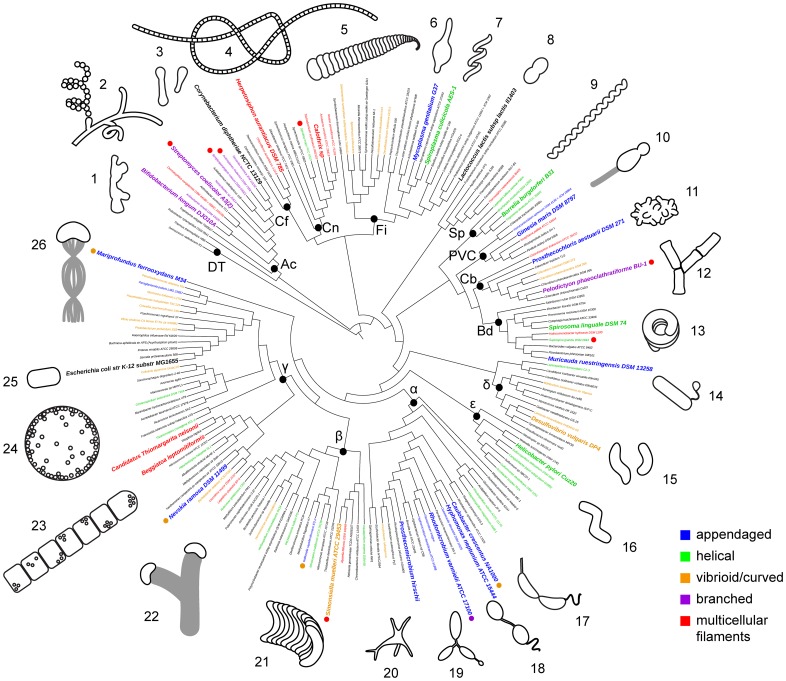
Myriad morphologies have evolved throughout the bacterial domain. Bacterial phylogeny derived from genome sequence data for selected species, with an emphasis on morphologically and phylogenetically diverse taxa. Sequence data gathered from the Joint Genome Institute [3] and the National Center for Biotechnology Information [4] were searched for reference genes and aligned using Phylosift [5]. FastTree [6] generated an approximate maximum likelihood tree from the resulting concatenated alignment. The final tree was formatted using iTol [7]. Black dots denote ancestral nodes of selected major taxa: **DT**, Deinococcus-Thermus; **Ac**, Actinobacteria; **Cf**, Chloroflexi; **Cn**, Cyanobacteria; **Fi**, Firmicutes (inclusive of Mollicutes); **Sp**, Spirochetes; **PVC**, Planctomycetes, Verrucomicrobia, Chlamydiae; **Cb**, Chlorobi; **Bd**, Bacteroidetes; **α**, **β**, **γ**, **δ**, **ε**, Proteobacteria subdivisions. **1. *Bifidobacterium longum*. 2. *Streptomyces coelicolor*** (mycelial [multicellular] filament with hyphae and spores). **3. *Corynebacterium diphtheria*e** (two cells, dumbbell and club shapes). **4. *Herpetosiphon aurantiacus*** (filament of multiple cylindrical cells). **5. *Calothrix*** (filament of multiple disk-shaped cells). **6. *Mycoplasma genitalium*. 7. S*piroplasma culicicola*. 8. *Lactococcus lactis*** (predivisional cell). **9. *Borrelia burgdorferi***. **10. *Gimesia maris*** (previously *Planctomyces maris*, predivisional cell with proteinaceous stalk). **11. *Prosthecochloris aestuarii***. **12. *Pelodictyon phaeoclathratiforme*** (filament of multiple trapezoidal cells). **13. *Spirosoma linguale***. **14. *Muricauda ruestringensis*** (appendage includes nonreproductive bulb). **15. *Desulfovibrio vulgaris*** (two cells, helical and curved shapes). **16. *Helicobacter pylori***. **17. *Caulobacter crescentus*** (predivisional cell). **18. *Hyphomonas neptunium*** (predivisional cell)**. 19. *Rhodomicrobium vannielii*** (filament of multiple ovoid cells, one is predivisional)**. 20. *Prosthecomicrobium hirschii***. **21. *Simonsiella muelleri*** (filament of multiple curved cells). **22. *Nevskia ramosa*** (two cells with bifurcating slime stalk)**. 23. *Beggiatoa leptomitiformis*** (filament of multiple, giant cylindrical cells). **24. *Thiomargarita nelsonii*** (single, giant cell). **25. *Escherichia coli*. 26. *Mariprofundus ferrooxydans*** (single cell with metal-encrusted stalk). Bacterial schematics are not to scale. Species names are colored according to morphology as indicated in the key. Colored dots are appended to indicate species with multiple morphologies. Names of species depicted in schematics are emphasized in large, bold font.

Or did you imagine a rod; in particular, one that elongates to double its length and then divides in two?

Perusing the once-definitive guide to bacterial identification, *Bergey’s Manual of Determinative Bacteriology*, one easily finds shapes much more interesting than rods and cocci [8]. Even the language used to describe the morphologies of various species in the text quickly illustrates the veritable bacterial zoo found on earth: In addition to the familiar coccoid, rod-shaped, or spirillar types, there are also dendroid, coryneform, cylindrical, bulbiform, fusiform, and vibrioid types. There are uniseriate or multiseriate filaments of cells that are flexible or rigid, flat or round, unbound or bound in hyaline or slime sheaths. Single cells are described as star-shaped, disk-shaped, hourglass-shaped, lemon-shaped, pear-shaped, crescent-shaped, or flask-shaped. Rods can be pleomorphic, straight, curved, or bent, with blunt, pointed, rounded, or tapered ends. Some cells grow appendages such as prosthecae, stalks, or spikes. The representative schematics in Fig 1 offer a glimpse of some of this diversity but hardly do justice to the variation of size and shape across the bacterial domain. *Bergey’s* served as a guide for identifying species phenotypically for a century, underscoring how reliably each species reproduces its signature morphology.

A curious reader of *Bergey’s* may find it perturbing that the more unusually shaped bacteria comprise a minority of the book, and most of the micrographs and notes on them date from before 1980. If morphological diversity is so pervasive, why do rods and cocci dominate the manual? And why is the information so old? These deficiencies not only reveal the historical focus of the field of microbiology on pathogenic bacteria, which tend to be rods and cocci, but also the shift in interest of the field to model organisms on the advent of molecular biology. During the 1970s, significant progress was made in gaining genetic control over *Escherichia coli*, thereby establishing it as the model bacterium [9,10]. Since that era, model bacteria such as *E*. *coli* and *Bacillus subtilis* have dominated research because of their genetic tractability and culturing ease. Many of the more strangely shaped bacteria proved unculturable, or their original strains were lost. In effect, *Bergey’s* serves as some sort of time capsule from which it is clear that a great diversity of bacterial morphologies exists. Sadly, this diversity is still likely to be highly undersampled, as the high-throughput metagenomic approaches that are quickly filling out the bacterial domain do not capture morphological data. A more complete visual survey of the bacterial domain would reveal more morphologies, the number of species with atypical morphotypes might rival those of the known rods and cocci, and those “typical” rods and cocci would exhibit a great deal more morphological variability than currently projected by the field. How are these diverse morphologies related evolutionarily and mechanistically, and what are their functions?

## Morphology and Bacterial Evolution

Phylogenetic trees based on molecular sequence data have transformed how we understand bacterial evolutionary relationships [11]. Such phylogenies have proven that the historical taxonomic approach used to classify bacteria based on phenotypes such as morphology often grouped bacteria unrelated by descent. For example, the Betaproteobacteria *Rhodocyclus tenuis* and *Rubrivivax gelatinosus* were misclassified as members of the genus *Rhodospirillum*, and therefore as Alphaproteobacteria, partially on the basis of their helical shape [12]. Clearly, even careful and expert interpretation of phenotypes alone can lead to misinterpretations of relatedness between species. In another case, appendages called prosthecae, which are thin extensions of the entire cell envelope, were considered a unifying characteristic of the genus *Prosthecomicrobium*. However, 16S phylogenetic analysis led to the split of the single genus into three separate genera that were each more closely related to a nonprosthecate genus than to one another [13]. Thus the prosthecate morphology most likely was shared by a common ancestor to this group and was lost in some lineages. Together, these examples suggest that (1) similar-looking morphologies can and do evolve independently in unrelated genera and that (2) the histories of certain morphologies among related genera can be complex.

Mapping our current, yet limited, knowledge of morphological phenotypes onto robust phylogenies allows us to make inferences about how bacterial shapes evolved. This approach has seen extensive application for inferring the evolutionary history of traits in eukaryotes [14], and it was similarly applied to exploring transitions from rod to coccus shapes in the bacterial domain [15]. Using updated phylogenetic tools, we have constructed a phylogeny that focuses on diverse morphologies beyond rods and cocci (Fig 1). Some morphologies, such as helical cells (green) or multicellular filamentous types (red), appear repeatedly throughout the bacterial domain. This scattering amongst distant clades indicates that these morphologies have several, independent evolutionary origins. In some cases, the same morphology appears to cluster in a specific region of the phylogenetic tree, such as branching in the Actinobacteria (purple) or appendages in the Caulobacterales (blue). This clustering indicates persistence of a morphology inherited from a shared ancestor. These relationships between phylogeny and morphology generate many questions important to evolutionary biology and bacteriology. When the same shape arises independently, different lineages with distinct constraints somehow arrive at a common morphology: Are these shapes generated via similar molecular strategies? Are they influenced by similarities in the environment or bacterial lifestyle? In cases of shared ancestry, we can address the persistence of a specific morphology: Is the shape retained through continued selective pressure? How and why does morphological variation among family members arise? We are only beginning to answer these questions. However, our developing perspective on bacterial diversity and the ability to map morphologies onto the ever-growing phylogenetic tree of the bacterial domain will allow us to better address them.

The reason that bacteria have certain shapes remains unclear; we can only surmise that certain shapes have adaptive value or have been produced by other selective forces since they are under genetic control and are maintained from generation to generation. Many theories offer rationales for the adaptive value of specific morphologies [16,17]. Young’s [16] excellent review explores the relationship of selective forces and bacterial morphology in detail that goes well beyond the scope of this essay. In general, morphological traits can be attributed to adaptation to selective forces such as nutrient limitation, reproduction, attachment, dispersal, and evasion of predation or host detection. In the simplest formulations, helical and curved cells appear to be optimized for motility, especially in viscous solutions [18–21], large cells (and very small cells) for evading ingestion (or capture) by protists [22,23], and branching and net-forming cells for buoyancy and soil retention [24,25].

We can easily speculate on the adaptive value of the morphologies presented in Fig 1. Take for example the extremely long (100–1,200 μm) multicellular filaments of *Herpetosiphon aurantiacus* (Fig 1.4), which are capable of rapid gliding motility and remarkable flexibility [26,27]. It is possible that the extraordinary lengths of these filaments allow this bacterium to evade phagocytosis by protists or boost its gliding motility on surfaces. Perhaps the flexibility of the multicellular filaments allows entwinement with various substrates in aqueous or soil environments to enhance adherence and retention. Even more exciting, these traits may play an important role in its facultative predation of other bacteria, in which its looped filaments trap and “bulldoze” other bacteria, lysing them with secreted hydrolases [28]. Or, consider the case of *Pelodictyon phaeoclathratiforme* (Fig 1.12), a filamentous green sulfur bacterium that forms three-dimensional nets of trapezoidal cells via branching and possibly ternary division [29,30]. Arguably, these nets slow sedimentation and help to keep colonies of this bacterium at the correct level in the water column, where they are optimally situated between opposing gradients of light and sulfide [31]. Or, take for example the extreme size of members of the genus *Thiomargarita* (Fig 1.24), a coccus that averages 0.1–0.3 mm in diameter [32–34]. This bacterium breaks the usual limitations of diffusion on size by harboring a nitrate-rich, liquid-filled vacuole that takes up 80%–98% of the cell volume and leaves only a 0.5–2 μm layer of cytoplasm [32]. The large size of *Thiomargarita* has been posited as an adaptation for a nonmotile lifestyle in which periodic resuspension of the large, buoyant cells puts them in contact with the oxygenated water column [33,34]. The vacuole, as well as a host of cytoplasmic sulfur inclusions, may also serve as a nutrient reservoir, which enables the bacterium to maintain metabolism during nutrient limitation between resuspension events.

Adaptive explanations for bacterial morphology provide a convenient narrative, but we must also account for alternatives. Consider the proposed roles of *Herpetosiphon*’s multicellular filamentation: gliding motility, predation defense, and its own predatory behavior. The shape may serve an adaptive role in just one of these functions or some combination thereof. However, all these guesses may be off the mark. Perhaps multicellular filamentation instead provides a different function, such as clonal cohesion in order to reinforce cooperative behavior like nutrient scavenging [35]. Or, the observed morphology may arise merely by chance rather than adaptation [36]. Alternatively, multicellular filamentation may arise as a byproduct of selection for a different phenotype [37], such as enhanced surface attachment that also happens to increase attachment between cells. All too often, adaptive explanations for morphology comprise quaint, just-so stories, residing unchallenged at the end of an article’s discussion section. Validating adaptive hypotheses for morphology requires the same scrutiny applied to other scientific problems: generating and testing a clear, falsifiable hypothesis. Only with clear, empirically tested hypotheses regarding the selective function of shape can we begin to paint a clearer picture regarding the environmental pressures that have shaped the historical evolution of the diverse array of observed bacterial morphologies.

## Mechanisms of Morphogenesis

Although it can be very difficult to determine why bacteria have certain morphologies, many inroads have been made into determining how they obtain them. The vast majority of bacteria synthesize a peptidoglycan (PG) cell wall that provides structural integrity to the cell. The growth, maintenance, and modification of the cell wall play a key role in defining the shape of the cell [38]. Much of what we know regarding bacterial shape determination at the molecular level in bacteria comes from the study of a few model organisms with relatively simple morphologies, including the following: the sphere or ovoid shape of *Streptococcus pneumoniae* or *Staphylococcus aureus*; the rod shape of *E*. *coli*, *B*. *subtilis*, or *Agrobacterium tumefaciens*; and the spiral shape of *Helicobacter pylori*. These model systems have proven experimentally advantageous in understanding basic and common principles involved in different modes of bacterial cell growth. A conserved set of proteins participates in PG synthesis and remodeling [39,40], thus the details of cell wall synthesis gathered from select model systems provide a solid touchstone for exploring even divergent morphologies.

**Fig 2:**
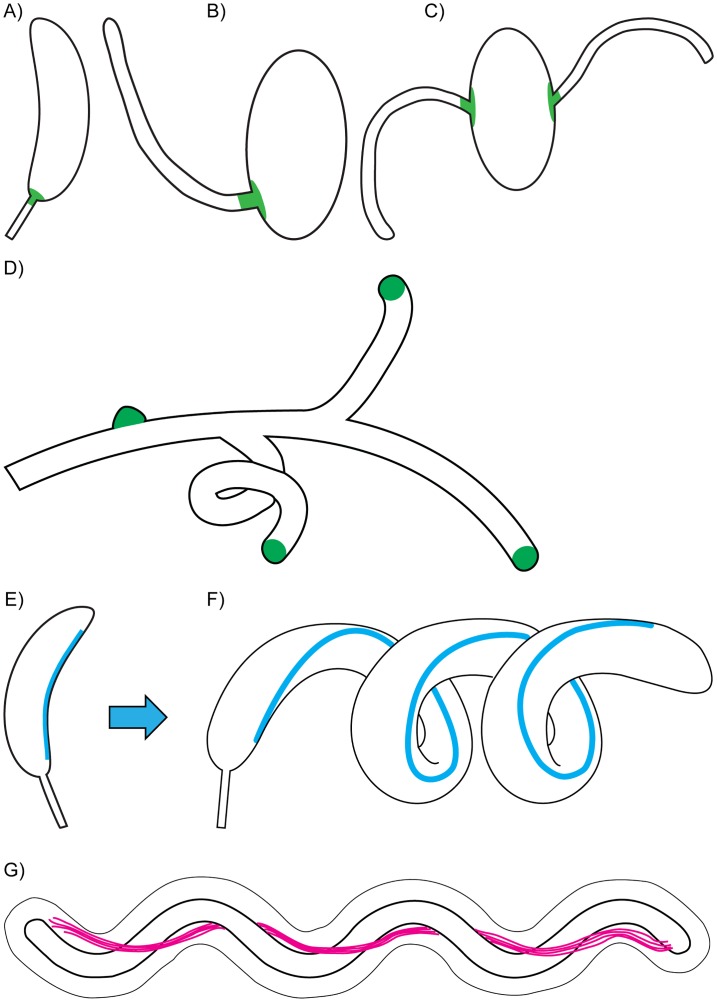
Different mechanisms underlie the evolution of morphogenesis. **(A–C)** In prosthecate Alphaproteobacteria, simply repositioning zonal PG synthesis machinery (shown in green) can generate polar (*Caulobacter crescentus*, **A**), subpolar (*Asticcacaulis excentricus*, **B**), or bilateral (*Asticcacaulis biprosthecum*, **C**) prosthecate phenotypes. **(D)** Similar repositioning of the PG growth zone yields a branching phenotype in *Streptomyces coelicolor*, which grows from the cell poles. **(E)** The intermediate filament-like protein crescentin (shown in cyan) of *C*. *crescentus* constrains PG synthesis to generate cell wall curvature. **(F)** When cells filament, this constraint results in long helical shapes. **(G)** The periplasmic flagella of *Borrelia burgdorferi* directly deform the cell body into a planar wave shape. Note that the scale of the periplasmic space, relative to the cell membranes, has been modified to highlight this arrangement. For simplicity, details of the periplasmic compartment are shown only for panel **G**.

Bacteria must remodel the cell wall in order to grow and divide, and this activity underlies their shape-generating capacity. Because PG synthesis is constrained in space, all cell wall growth and remodeling can be described as “zonal” growth at the molecular level [41]. Zonal growth at specific points during the cell cycle gives rise to specific patterns of PG synthesis, or growth modes. A number of growth modes have been shown to generate the rod shape. The best-studied growth mode, exemplified by *B*. *subtilis*, is lateral elongation, in which PG synthesis is evenly dispersed along the length of the cell. *E*. *coli* and *Caulobacter crescentus* utilize lateral elongation as well as an additional growth mode called preseptal or medial elongation, in which PG synthesis occurs at the midcell. Alternatively, some bacteria, such as *A*. *tumefaciens*, employ polar elongation at one or both poles [42]. Despite differences in the specific details of each system at the molecular level, all these growth modes can be viewed as a modular, spatiotemporal utilization of zonal growth to create the rod shape. But how does zonal PG synthesis generate diverse shapes beyond simple rods and cocci? Conceptually, simply repositioning zonal growth to specific locations could generate a diverse range of morphologies derived from the basic rod shape. Take as an example the *Caulobacter* prostheca, which results from zonal growth restricted to the cell pole (Fig 2A). In the closely related genus, *Asticcacaulis*, distinct prosthecate morphologies arise from repositioning the growth zone(s) at subpolar and bilateral sites (Fig 2B and 2C) [43,44]. In Actinobacteria, branching results from repositioning the polar growth PG machinery to create new growing poles (Fig 2D) [45]. Thus, simple variations on the theme of zonal PG growth can give rise to diverse morphologies.

While similar growth mechanisms may yield diverse morphologies among close relatives, can shared mechanisms also explain similar morphologies in phylogenetically distant bacteria? An interesting comparison can be made between the alphaproteobacterium *C*. *crescentus* and the spirochete *Borrelia burgdorferi*, which are only very distantly related. *C*. *crescentus* exhibits a vibrioid (curved) morphology, which gives rise to helical shapes when the cells filament (Fig 2E and 2F) [46]. This morphology requires the species-specific molecular scaffold crescentin, which imposes a structural constraint along the inner curve of the cell to guide PG synthesis [46,47]. In contrast, cell curvature in the spirochete *B*. *burgdorferi* follows different rules, relying on a structural role of periplasmic flagella. In this case, what may appear to be a helical morphology instead comprises a “flat wave” shape created by the constraints of the periplasmic flagella (Fig 2G). Rather than inducing curvature via differential PG synthesis, the periplasmic flagella are posited to directly deform the cell body to generate curvature [48]. Despite their mechanistic differences, both *C*. *crescentus* and *B*. *burgdorferi* generate curvature according to a common structural theme: scaffold-mediated delineation of an inner curvature. It will be interesting to see whether other examples of evolutionarily distinct curvature and helicity depend on similar themes. Do all curved cells require a scaffold? Do such scaffolds share particular physical or biochemical properties? Indeed, cytoplasmic scaffolding proteins (in addition to PG-modifying enzymes [49,50]) participate in helical morphogenesis in *H*. *pylori* [51,52]. Like crescentin, these proteins self-oligomerize and contain coiled-coil motifs known to participate in protein–protein interactions. Although detailed experimentation is required in order to identify the components used to achieve morphogenesis in particular species, unraveling these mechanistic details can help to identify general morphogenetic strategies that may reveal fundamental constraints on how particular shapes evolve. These general trends can guide our investigations of morphogenetic mechanisms in other bacteria and enhance our understanding of how bacterial shape evolves.

## A Path Forward

Although microbiologists have long appreciated the impressive morphological diversity among bacteria, we still have much to learn. Modern technologies provide access to unprecedented quantities of data, particularly microbial genome sequences. However, harnessing these data to address the “how” and “why” of morphological evolution presents a challenge.

We must first consider where to direct our efforts in order to learn about morphology. A clear picture of morphological evolution requires a representative sample of phylogenetic diversity thorough enough to identify important morphological transitions. This picture becomes muddied through oversampling of pathogens and other species of applied significance [53]. Repeatedly studying similar, closely related bacteria effectively resamples the same ancestral events [54]. Phylogenetically informed sampling helps to resolve this problem by identifying unique morphological transitions in the bacterial tree. More fundamentally, overemphasis on particular taxa limits our perspective on representative morphological variation across the bacterial domain. Recent genome sequencing efforts emphasize more phylogenetically comprehensive sampling, particularly the Genomic Encyclopedia of Bacteria and Archaea [55]. This approach has helped to span various independent morphological transitions among available genome sequences (Fig 1). Importantly, much of the bacterial domain remains uncharacterized [56], and numerous morphological variants likely await discovery. High-throughput culturing [57] generates relatively unbiased bacterial libraries from an environment of interest. High-content imaging [58] and automated image analysis provides an unbiased quantification of morphological parameters [59–61] to identify bacteria of morphological interest. Even among the very large fraction of bacteria that remain unculturable, the advent of single-cell genomics opens the door to sequencing genomes of morphologically relevant species [62]. This approach has already successfully paired high-resolution microscopy with whole-genome sequences of noncultured bacteria [63]. These strategies will help to provide a more complete picture of morphological diversity and characterize key morphological transitions that best inform our understanding of how bacterial shape evolves.

**Fig 3:**
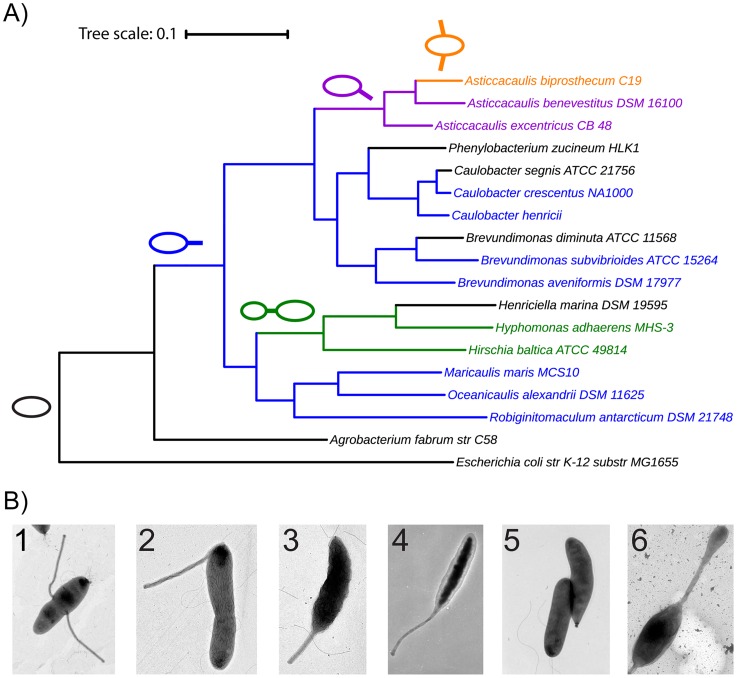
The Caulobacterales lineage exhibits diversification of the prosthecate morphology. **(A)** Phylogeny of the order Caulobacterales generated as described in Fig 1. Schematics and corresponding colors indicate inferred ancestral morphologies and their subsequent inheritance. Black branches indicate rod-shape, nonappendaged morphology, including several apparent prostheca loss events. Scale bar indicates 0.1 amino acid substitutions per site. **(B)** Transmission electron micrographs of members of the Caulobacterales, highlighting disparate prosthecate morphologies. For each morphology, a brief description and the name of one representative species is provided, followed by the image source in parentheses. **1. Bilateral prosthecae, *Asticcacaulis biprosthecum*** (Chao Jiang, Stanford University). **2. Subpolar prostheca, *Asticcacaulis excentricus*** (Chao Jiang, Stanford University). **3. Polar prostheca, *Caulobacter crescentus*** (Paul Caccamo, Indiana University). **4. Polar prostheca, *Maricaulis maris*** (Patrick Viollier, University of Geneva). **5. Short polar prostheca, *Brevundimonas subvibriodes*** (Brynn Heckel, Indiana University); note other members of this genus display a much longer prostheca. **6. Polar prostheca through which budding reproduction occurs, *Hirschia baltica*** (Paul Caccamo, Indiana University). Magnification varies between micrographs. All images are reproduced with permission.

Our laboratory used such a phylogenetically informed approach in order to understand the molecular basis of the prosthecate morphology in the model organism *Caulobacter* and closely related genera. The phylogeny of *Caulobacter*, *Brevundimonas*, and *Asticcacaulis* (Fig 3) indicates that an ancestral polar prostheca has been repositioned (first subpolar and then lateral in *Asticcacaulis*), increased in number (*Asticcacaulis biprosthecum*), and lost outright (*C*. *segnis*, *Brevundimonas diminuta*). We recently examined the morphological transition from a polar prostheca to subpolar or lateral prosthecae. Specifically, we determined a morphogenetic function for the protein SpmX, previously shown to participate in developmental regulation in *C*. *crescentus* [64]. In *Asticcacaullis*, SpmX has been co-opted to position and coordinate prostheca PG synthesis [43]. An expanded region within this protein appears to be responsible for the difference in localization patterns between *A*. *excentricus* (subpolar) and *A*. *biprosthecum* (bilateral). This particular story of morphogenesis mechanism and evolution emerged from a combination of two methods: comparative study of various *Asticcacaulis* and *Caulobacter* species and direct genetic manipulation informed by the well-developed *Caulobacter* experimental model. In this case, progress was possible because we utilized a model organism and its close relatives. However, many morphologically interesting species are poorly studied, with little in the way of prior research or available genetic tools. Is the study of bacterial morphology then stuck relying on a limited selection of existing, extensively developed experimental models and their close relatives? Thankfully, things are not so grim; comparative molecular genetics, automated image analysis, and advanced labeling tools all offer interesting solutions for studying morphology in underrepresented systems.

Genome sequence data can provide significant insight into the evolution of morphology. From sequence data, comparative molecular genetics can identify patterns of gene content, selection on individual genes (including individual residues), and functionally connected suites of genes [65,66]. Applying this approach to existing genome sequence data may reveal the selective targets associated with specific morphologies. Indeed, this comparative genomic strategy identified specific proteins associated with the transition from rods to cocci in pathogenic bacteria [67]. Of course, genomic data cannot substitute for testing morphological mechanisms by direct experimentation. Nonetheless, comparative genomics can help to identify suitable targets for empirical study.

Modern tools also facilitate direct morphological experimentation, even for species without previous experimental development. In particular, recently described fluorescent D-amino acids (FDAAs) label sites of cell wall synthesis in diverse bacteria by leveraging the ubiquity of PG [68,69]. This labeling strategy enables precise, microscopic observation of the growth modes underlying interesting morphologies, even for bacteria that are poorly understood or experimentally intractable. Another valuable tool, high-throughput imaging, can identify morphological determinants even in the absence of advanced molecular genetic tools. Imaging mutant libraries can identify relevant protein targets by screening for specific phenotypes such as the following: (1) morphological variants in libraries generated by transposon or chemical mutagenesis and (2) proteins localized to morphological features in fluorescent transposon fusion libraries. Finally, automated analysis of cell images provides efficient and quantitative analysis of large mutant libraries [59–61]. These tools permit relatively detailed analysis of bacterial morphogenesis in model and nonmodel organisms alike.

We propose an experimental strategy for leveraging these tools to study novel morphogenetic mechanisms in order to determine both how patterned cell growth generates specific morphologies and the underlying molecular machinery. Pulse labeling with FDAAs reveals the cell wall growth pattern responsible for generating a particular bacterial shape [68]. Such underlying growth patterns are not always obvious from the final cell shape, even for relatively simple shapes like rods [42]. After growth patterns have been determined, the next goal is to identify genetic loci that participate in morphogenesis. Mutant libraries can be generated through transposon or chemical mutagenesis and then screened by high content imaging and automated image analysis for variants in shape and FDAA labeling patterns. Sequencing of the mutated site (transposon) or of the whole genome (chemical mutagenesis) can then identify affected loci. This broadly applicable experimental strategy can reveal both underlying growth patterns and mechanistic components responsible for generating particular morphotypes. Applying this strategy across diverse taxa and morphologies can identify common themes and points of departure in mechanisms of morphogenesis. By casting a wide net, such observations can guide further development of key model organisms for careful inquiry at the molecular level.

Even as we unravel the mechanistic details of bacterial morphogenesis, the ultimate explanation for the varied shapes observed in nature derives from the selective forces at play in the environment. Complex morphologies like spirals and appendages do not emerge and persist for millions of years by chance alone. However, as discussed earlier, determining the adaptive value of shape requires moving beyond telling plausible, “just so” stories. Fortunately, evolutionary biology already provides a theoretical and experimental framework for directly examining how morphology influences bacterial fitness.

Compelling evidence of the adaptive value of shape requires direct observation of selection acting on phenotype. The most direct evidence of adaptive value derives from head-to-head competition of different morphotypes and the emergence of a superior competitor via selection. A recent study employed pairwise competition of distinct *E*. *coli* cell size mutants, identifying fitness effects of cell size that depend on the particular growth environment [70]. Similarly, direct competition among morphotypes of *H*. *pylori* has proven that native cell curvature enhances stomach colonization when compared against mutants with reduced curvature [49], demonstrating that shape is a key fitness component for a pathogen. Future work should follow a similar path in establishing adaptive relevance of morphology, varying both morphotype and environment while measuring fitness through direct competition.

We envision a combined strategy of direct competition assays and laboratory evolution for testing hypothesized functional significance of particular bacterial morphologies. Based on a hypothesized adaptation to a specific environmental constraint, competition assays should identify a selective advantage of the adaptive morphotype over its mutant competitors, but only when the constraint is imposed. Let us return to the example of *Pelodictyon*, whose branched cell networks hypothetically enhance fitness by retarding sedimentation and maintaining correct cell position along light and sulfite gradients. According to this sedimentation hypothesis, wild-type cells should readily outcompete a non–net-forming mutant in an appropriately spatially structured laboratory environment. However, agitation to disrupt spatial environmental gradients should abolish the wild-type competitive advantage conferred by retarded sedimentation. Failure of the wild type to outcompete the mutant in the structured environment would suggest an alternate adaptive role of the branched cell network morphology. Additionally, we can use experimental evolution to observe natural selection of morphology directly in the laboratory. If selection for reduced sedimentation maintains the filamentous network morphology of *Pelodictyon*, selection of *Pelodictyon* in agitated (unstructured) environments should result in the eventual erosion of the morphology via genetic drift, whereas continued selection in a structured environment should preserve the native phenotype. Although we cannot reconstruct historical environmental pressures with certainty, experiments like these can provide empirical support for adaptive hypotheses. This proposed strategy for testing the functional basis of morphology elevates adaptive hypothesis beyond throwaway comments in the discussion and into their rightful position among the results.

## Conclusion

Decades after an initial surge in environmental microbiology described a vast array of different bacterial shapes, we are left with an impressive body of knowledge describing what is out there, but relatively little in the way of how and why. While much remains unresolved regarding the details of bacterial morphogenesis, the current scientific landscape affords great opportunity for progress. By pairing abundant genome sequence data with modern tools for imaging cell growth, new experimental approaches are now available for morphologically diverse bacteria, even in cases in which little prior research exists. We hope this opportunity yields new insights and renewed interest in certain less familiar, morphologically varied bacteria that lie beyond rods and cocci.

## Supporting Information

S1 Fig High-resolution version of Fig 1. Vector graphics format version of Fig 1.
(PDF)

## Abbreviations

FDAA: fluorescent D-amino acid
PG: peptidoglycan
