# Supplementary figures and images for: Diversity Takes Shape: Understanding the Mechanistic and Adaptive Basis of Bacterial Morphology
Source: PLoS Biol. 2016 Oct 3;14(10):e1002565. doi: 10.1371/journal.pbio.1002565 (PMC5047622; doi:10.1371/journal.pbio.1002565)

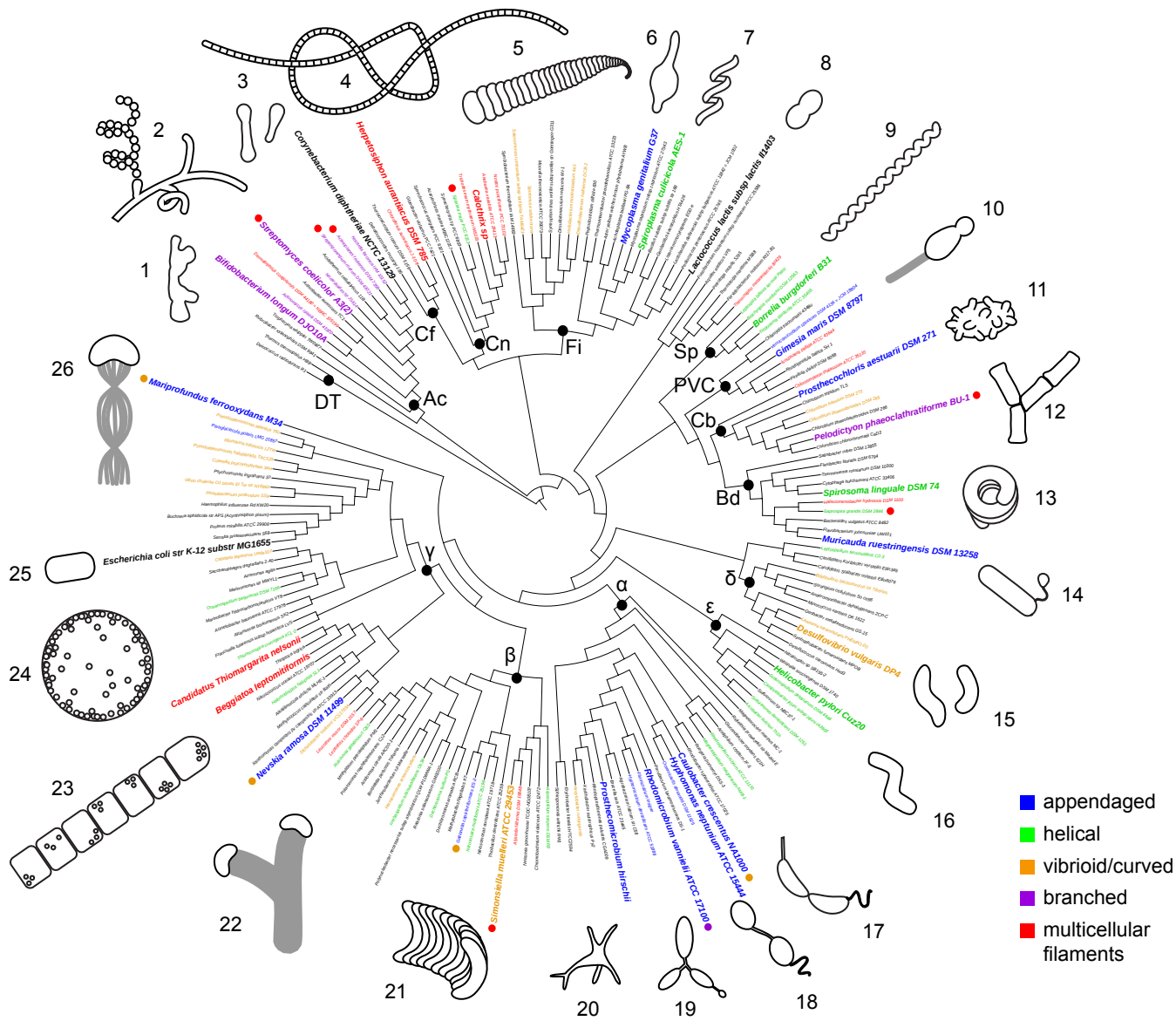

Supplement: S1 Fig — Vector graphics format version of Fig 1. (PDF) [file pbio.1002565.s001.pdf]
